# Enhanced parietal cortex activation during location detection in children with autism

**DOI:** 10.1186/1866-1955-6-37

**Published:** 2014-09-19

**Authors:** Thomas P DeRamus, Briley S Black, Mark R Pennick, Rajesh K Kana

**Affiliations:** Behavioral Neuroscience Graduate Program, Department of Psychology, University of Alabama at Birmingham, Birmingham, AL 35294-1170 USA; Undergraduate Neuroscience Program, University of Alabama at Birmingham, Birmingham, AL 35294-1170 USA; Lifespan and Developmental Psychology Graduate Program, Department of Psychology, University of Alabama at Birmingham, Birmingham, AL 35294-1170 USA; Department of Psychology, University of Alabama at Birmingham, Birmingham, AL 35294-1170 USA

**Keywords:** fMRI, Autism, Dorsal, Ventral, Visual system, Functional connectivity, Object recognition, Location detection

## Abstract

**Background:**

Visuospatial processing has been found to be mediated primarily by two cortical routes, one of which is unique to recognizing objects (occipital-temporal, ventral or “what” pathway) and the other to detecting the location of objects in space (parietal-occipital, dorsal or “where” pathway). Considering previous findings of relative advantage in people with autism in visuospatial processing, this functional MRI study examined the connectivity in the dorsal and ventral pathways in high-functioning children with autism.

**Methods:**

Seventeen high-functioning children and adolescents with autism spectrum disorders (ASD) and 19 age-and-IQ-matched typically developing (TD) participants took part in this study. A simple visual task involving object recognition and location detection was used. In the MRI scanner, participants were shown grey scale pictures of objects (e.g., toys, household items, etc.) and were asked to identify the objects presented or to specify the location of objects relative to a cross at the center of the screen.

**Results:**

Children with ASD, relative to TD children, displayed significantly greater activation in the left inferior parietal lobule (especially the angular gyrus) while detecting the location of objects. However, there were no group differences in brain activity during object recognition. There were also differences in functional connectivity, with the ASD participants showing decreased connectivity of the inferior temporal area with parietal and occipital areas during location detection.

**Conclusions:**

The results of this study underscore previous findings of an increased reliance on visuospatial processing (increased parietal activation) for information processing in ASD individuals. In addition, such processing may be more local, focal, and detailed in ASD as evidenced from the weaker functional connectivity.

**Electronic supplementary material:**

The online version of this article (doi:10.1186/1866-1955-6-37) contains supplementary material, which is available to authorized users.

## Background

The perception and interpretation of visual stimuli are vital for human beings navigating the world. Research on visual information processing has been of great interest to neuroscience, with one of the most widely accepted models of perception proposed in the early 1980s [1]. This model suggested two visual pathways in the brain, one in which visual information relating to object identification is processed (the “what” or “ventral” pathway) and the other in which spatial location of objects is processed (the “where” or “dorsal” pathway). The ventral pathway extends from the visual cortex into various temporal lobe structures associated with object stimuli and responds to features, patterns, faces, and color stimuli [2]. Lesion studies in humans and non-human primates have revealed that damage to the structures along this pathway can lead to deficits in object recognition and/or discrimination [2, 3]. In contrast, the dorsal pathway extends from the visual cortex towards parietal areas (including the precuneus, lingual gyrus, and parietal lobules) and is associated with the orientation and location of objects [2, 3]. Lesions to these areas can lead to visual neglect and spatial misrepresentations of body movements [3, 4]. The communication between dorsal and ventral pathways, mediated by connections with the frontal cortex, may be critical in visual information processing [5].

Neuroimaging studies have provided widespread support for the differential role of the dorsal and ventral processing streams in healthy control participants [4, 6–8]. However, investigations on the behavior of these networks in neurodevelopmental disorders, such as autism spectrum disorders (ASD) are sparse. This is especially interesting considering the relatively intact or superior visuospatial processing abilities reported widely in autism [9–12]. This has been reported across visuospatial cueing task [9, 13, 14], Embedded Figures Task [15, 16] block design task [17, 18], and visual search tasks [19, 20]. Additionally, fMRI studies have found greater activation within visuospatial areas and less activation in frontal areas in the ASD brain during such tasks [10, 21]. Evidence from these studies point to enhanced “lower level” (increased reliance on parietal/occipital areas) processing skills for objects in ASD individuals compared to typical control individuals. While this hypothesis has been frequently applied to face processing and feature detection within ASD, to our knowledge, there are no functional imaging studies specific to detecting objects and location in space in the ASD literature.

Intact visuospatial processing and increased posterior brain activity in autism have been found to be accompanied with intact or enhanced functional connectivity within these areas [21]. A relatively consistent finding in neuroimaging research in ASD is a decrease in functional connectivity between the frontal cortices and posterior (parietal and occipital) regions of the brain [22–25]. Such findings have led to the theory that individuals with ASD may have more isolated functional networks compared to typical controls [23], which may be reflected by the differential processing strategies. Another view is that the superior visuospatial processing may be a consequence of the frontal-posterior underconnectivity which results in a parietal autonomy in autism [26]. The primary goal of the present study was to use a relatively simple object recognition-location detection paradigm to examine the connectivity of the dorsal and ventral visual streams in autism. Based on the visuospatial superiority in autism, we hypothesized intact or enhanced connectivity in these streams in participants with autism. While it is uncertain if there will be differential activation of these networks in ASD, previous literature suggests that there should be increased occipital and parietal/temporal activity and decreased frontal activation within individuals with ASD [21, 27]. While dorsal and ventral stream connectivity has been examined in adults with autism before [22], our study is novel in examining dorsal-ventral connectivity in children with autism. This is an important avenue in the context of behavioral findings of visuospatial advantage reported in individuals with autism. Thus, the findings of this study will provide insights into the neural circuitry underlying one of the basic mechanisms of visual processing in autism.

## Methods

### Participants

Seventeen high-functioning children and adolescents with ASD (mean age: 13.45 years, SD: 1.73; male/female: 16/1) and 19 typically developing (TD) control participants (mean age: 12.41, SD: 1.56; male/female: 16/2) took part in this fMRI study of object recognition and location detection. Both groups were matched on age, and IQ, measured by the Wechsler Abbreviated Scale of Intelligence (WASI) (see Table 1 for demographic information).

**Table 1:** **Participant demographic information**

	ASD (*n* = 17)			TD (*n* = 19)			Group difference	
	Mean	Range	SD	Mean	Range	SD	*t*-value	*p*value
Age	13.45	10.8–17.1	1.73	12.41	10.3–15.5	1.56	-1.86	0.07
VIQ	102.3	79–128	15.41	107.4	83–134	15.71	0.33	0.31
PIQ	102.7	74–132	13.3	104.5	73–137	13.48	1.02	0.75
FSIQ	102.8	75–126	18.8	106.8	81–139	15.51	0.76	0.45

*PIQ* performance IQ, *VIQ* verbal IQ, *FSIQ* full scale IQ.

Scans were acquired for 22 TD and 21 ASD participants. This study was approved by the Institutional Review Board of the University of Alabama at Birmingham, and all participants provided informed consent for their participation in the study. Each participant’s data were examined for continuous head motion, intermittent spikes, and drifts in *x*, *y*, and *z* translational directions after the realignment during data preprocessing, and stringent head motion criteria were applied to ensure data quality. Participants were excluded if more than 20% of their functional images displayed excessive motion (more than 2 mm) in any direction. These criteria resulted in a total of 19 TD and 17 ASD participants. Any remaining motion within each subject’s scans was entered into the general linear model in SPM8 (Wellcome Department of Cognitive Neurology, London, UK) as a regressor of no interest. A two-tailed, independent sample Mann-Whitney *U* test was performed using IBM SPSS 22 (using the TD group as the reference group) to determine the distribution of head motion across six dimensions (translation: *x*, *y*, and *z*; rotation: pitch, roll, and yaw). This analysis did not reveal any statistically significant difference in head motion between the two groups in any of the dimensions: *x*: *U*(33) = 144, z = -0.297, *p* = 0.766; *y*: *U*(33) = 140, *z* = -0.165, *p* = 0.668; *z*: *U*(33) = 148, *z* = -0.165, *p* = 0.869; *pitch*: *U*(33) = 146, *z* = -0.817, *p* = 0.817; *roll*: *U*(33) = 139, *z* = -0.462, *p* = 0.644; and *yaw*: *U*(33) = 140, *z* = -0.429, *p* = 0.668.

### Task

We used a simple object recognition and location detection paradigm to examine functional connectivity in participants with autism. A series of grey-scale photographs of small common household objects overlaid against a black background were presented in the MRI scanner in a blocked design format. The objects presented included, but were not limited to, the following categories: miniature animals, children’s toys, kitchen objects, and clothing items. These stimuli were adapted from Pennick and Kana [8]. Each stimulus item presented during the experiment was unique, and the presentations of the blocks were pseudorandomized with two tasks (four blocks of object recognition and four blocks of location detection) and five iterations of a fixation baseline each lasting 24 s. During the object recognition blocks, participants were asked to identify a given object by choosing the name of the object from a list of four answer choices. During the location detection block, participants identified the location of a given object relative to a fixation cross at the center of the screen; the answer choices (left, right, above, below) indicated the object’s location relative to the cross. Responses were recorded using fiber optic buttons. The recorded responses provided the reaction time and performance accuracy data for the object recognition and location detection tasks. For both tasks, each stimulus was presented for 6 s, during which the participant chose an answer. Each block consisted of six pictures with an inter-stimulus interval of 1 s.

### Data acquisition and analysis

All participants practiced the experiment on a laptop computer before the scanning session. While in the scanner, E-Prime 1.2 (Psychology Software Tools, Inc., Pittsburgh, USA) was used to present the stimuli. An integrated functional imaging system (IFIS) interface projected the data onto a screen behind the participant’s head which was viewed using a mirror. Images were acquired using a 3 T Siemens Allegra head-only scanner housed at the Civitan International Research Center, University of Alabama at Birmingham. Structural images were acquired using high resolution T1-weighted scans using a 160 slice 3D MPRAGE volume scan with a repetition time (TR) = 200 ms, echo time (TE) = 3.34 ms, flip angle = 7°, field of view (FOV) = 25.6 cm, 256 × 256 matrix size, and 1-mm slice thickness. To record functional imaging data, a single-shot gradient-recalled echo-planar pulse sequence was used, which offers the advantage of rapid image acquisition (TR = 1,000 ms, TE = 30 ms, flip angle = 60°, FOV = 24 cm, matrix 64 × 64). This sequence covers most of the cortex (seventeen 5-mm-thick slices with a 1-mm gap were acquired in an oblique-axial orientation) in a single cycle of scanning (TR = 1) with an in-plane resolution of 3.75 × 3.75 × 5 mm^3^.

The data were preprocessed and statistically analyzed using SPM8 (Wellcome Department of Cognitive Neurology, London, UK). Images were corrected for slice acquisition timing, motion-corrected, and normalized to the MNI template, resampled to 2-mm^3^ voxels, and smoothed with a 6-mm full width half maximum (FWHM) kernel. Statistical analyses were performed on individual data using the general linear model, while group analysis used random-effects models. Areas of statistically significant activation were determined using *t*-statistics on a voxel-by-voxel basis. For statistical significance, the data were examined using family-wise error-corrected multiple comparisons (*p* < 0.05) for the contrasts between the tasks with fixation. For direct contrasts between conditions, we applied Monte Carlo simulations to the data using 3dClustSim in AFNI [28] within an average oblique-sliced mask generated from each subject’s functional images to determine the minimum number of voxels in each cluster threshold of *p* < 0.05. Based on this simulation, an uncorrected threshold of *p* = 0.005 and a cluster extent threshold of eighty-two 2-mm^3^ voxels were used for within and between-group comparisons.

### Functional connectivity analysis

Functional connectivity (FC; the synchronization of brain activation between regions) was computed (separately for each participant) as a correlation between the average time course of the signal intensity of all activated voxels from a given region of interest (ROI) with the average time course of the signal intensity of all activated voxels from every other ROI. The ROIs for FC analysis were defined by examining the clusters of significant activation for all participants (ASD + TD) for the contrast object + location vs. fixation so that it best represented all regions activated for object and location tasks. A total of 15 ROIs were defined which included the following: the supplementary motor area (SMA), bilateral inferior parietal lobule (LIPL, RIPL), thalamus (LTHAL, RTHAL), inferior temporal gyrus (LITG, RITG), superior parietal lobule (LSPL, RSPL), occipital cortex (LOC, ROC), left middle frontal gyrus (LMFG), left precentral gyrus (LPRCN), medial prefrontal cortex (MPFC), and right hippocampus (RHIP) (See Additional file 1: Figure S1 for the locations of these ROIs). Statistical *t*-maps from contrasts of the normalized and smoothed images were high-pass filtered and had the linear trend removed. Activation values from the *t*-maps that did not exceed a *t*-threshold of 3.0 were not included in statistical comparisons of the correlations. The time courses across the ROIs were correlated, and Fisher’s *r* to *z* transformation was applied to the correlation coefficients prior to averaging and performing statistical comparisons. In addition to the functional connectivity analysis across 15 ROIs, a connectivity network analysis was conducted by grouping 12 of the 15 ROIs into different networks based on the lobes and hemispheres to which they belong. These networks and the ROIs of which that they consist are: *left parietal* (LIPL, LSPL), *right parietal* (RIPL, RSPL), *inferior temporal* (LITG, RITG), *occipital* (LOC, ROC), and *frontal* (LMFG, LPRCN, SMA, MPFC). In addition to significantly reducing the number of statistical comparisons in connectivity analysis, the network analysis also allowed to test the functional connectivity among different lobes in mediating object recognition and location detection.

## Results

### Behavioral data

Performance accuracy and reaction time data collected in the scanner were analyzed to determine group differences and condition effects. Two-sample *t*-tests revealed no significant group differences for mean reaction time (location detection: TD mean = 2590.16 ms, ASD mean = 2512.24 ms; *t*(33) = 0.397; *p* = 0.397; object recognition: TD mean = 3135.25 ms, ASD mean = 3016.91 ms; *t*(33) = 0.704; *p* = 0.092) or for performance accuracy (location detection: TD mean = 70.8%, ASD mean = 63.7%; *t*(33) = 0.562; *p* = 0.931; object recognition: TD mean = 79.2%, ASD mean = 64.7%; *t*(33) = 0.092; *p* = 0.704) in either experimental condition. For within-group effects, paired sample *t*-tests revealed a significant effect of condition in TD participants for reaction time (location detection: mean = 2590.16 ms; object recognition: mean = 3135.25 ms; *t*(17) = 6.51; *p* < 0.001), with more time needed for object recognition relative to location detection task. There was no significant effect of condition on accuracy of trials with responses in TD participants (location detection: mean = 70.8%; object recognition: 79.2%; *t*(17) = -1.39; *p* = 0.183). In the ASD group, there was also a statistically significant effect of condition on reaction time (location detection: mean = 2512.24 ms; object recognition: mean = 3016.91 ms; *t*(16) = -4.47; *p* < 0.001), but not for accuracy to trials with responses (location detection: mean = 63.73%; object recognition: mean = 64.7%; *t*(16) = -0.234; *p* = 0.838). However, it should be noted that individuals in the ASD group had a much larger incidence of non-responses or responses outside of the 7-s response time compared to controls (*t*(68) = -2.377; *p* = 0.017). This difference seems to have been primarily driven by the object condition, as the number of missing data points in this condition for participants with ASD approached significance (*t*(33) = -2.50; *p* = 0.061), while that for location detection did not (*t*(33) = -1.50; *p* = 0. 143).

### Brain activation

Both object recognition and location detection tasks (when contrasted with fixation baseline) primarily activated occipital and parietal-temporal areas, including inferior parietal lobule (IPL), superior parietal lobule (SPL), and inferior temporal gyrus (ITG) bilaterally in ASD and TD participants. In addition, there was increased activity in bilateral frontal areas (precentral and middle frontal) in both groups, particularly during the object recognition task. Hippocampal and IFG activation was unique to contrasts involving object recognition in each group (see Additional file 2: Tables S1 and S2 for a detailed list of regions activated with cluster size for location vs. fixation and object vs. fixation contrasts for each group).

**Figure 1:**
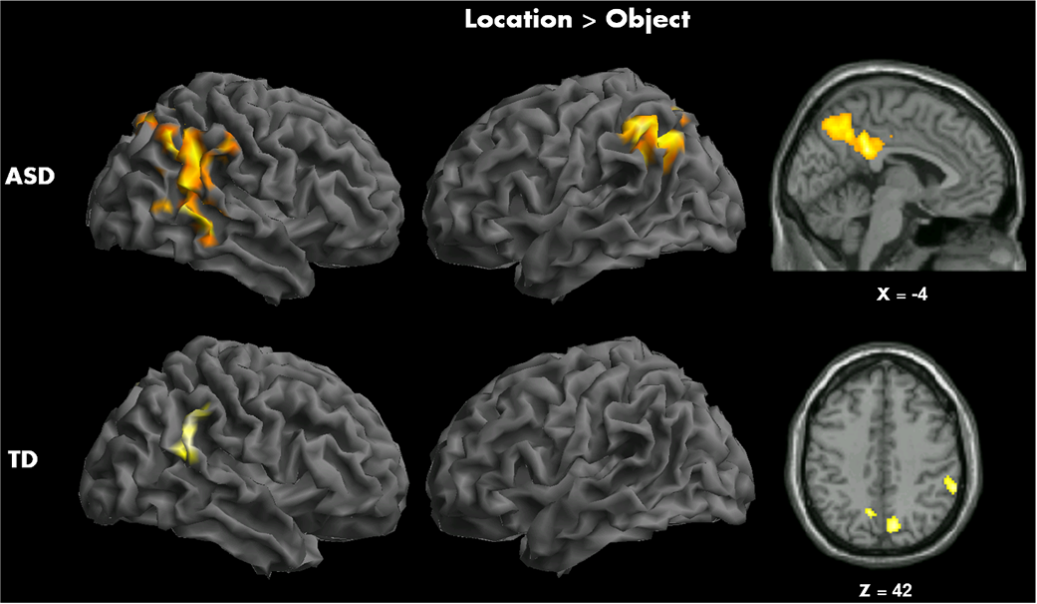
**Within-group activation maps for TD and ASD groups for location detection task contrasted with object recognition task.** *Top panel*: greater bilateral inferior parietal, precuneal, and middle/superior temporal regions in ASD participants; *bottom panel*: increased activation in right superior temporal and IPL areas in TD participants.

**Figure 2:**
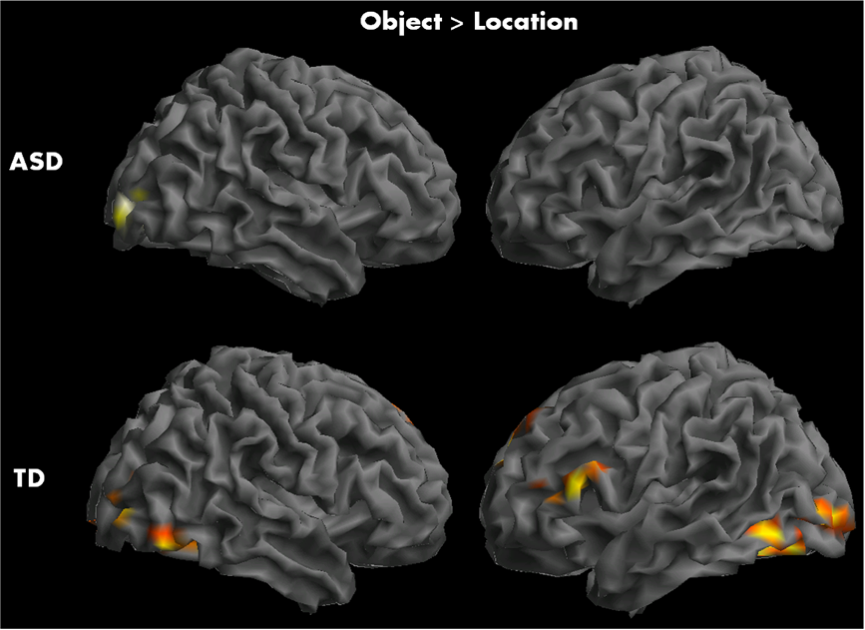
**Within-group activation maps for TD and ASD participants for object recognition task contrasted with location detection.** *Top panel*: increased activity in right calcarine and lingual gyrus in ASD participants; *bottom panel*: greater activity in bilateral inferior occipital, fusiform, inferior temporal, and frontal areas in TD participants.

Direct contrasts of location detection and object recognition within groups revealed robust bilateral IPL activation (location > object) in ASD participants along with precuneus and right middle/superior temporal cortex. The TD participants showed increased activation in left precuneus, right superior temporal, and IPL areas during this contrast (see Figure 1 and Table 2). Object recognition, when compared to location detection (object > location), elicited greater activity in bilateral inferior occipital, fusiform gyrus and ITG along with inferior, middle, and medial frontal areas in TD participants. The ASD participants, on the other hand, showed increased activity only in right calcarine and lingual gyrus in this contrast (see Figure 2 and Table 2) (*p* < 0.005 with a cluster size correction of 82 mm^3^).

**Table 2:** **Comparisons of activation between object and location tasks within ASD and TD groups**

	Hem	*x*	*y*	*z*	Cluster	*t*-value
Location > object						
ASD group						
Middle temporal	R	44	-64	6	1,919	5.82
Superior temporal	R	62	-40	4	1,919	5.42
Cingulate	R	-4	-40	30	2,226	5.34
Cingulate	L	-10	-34	32	2,226	5.23
Superior parietal	L	14	-48	34	2,226	5.06
Cingulum/precuneus	R	-40	-60	36	1,126	4.85
Angular	L	-50	-60	44	1,126	4.78
Angular	L	-44	-50	46	1,126	4.63
Middle frontal	R	32	18	44	141	3.88
Middle frontal/superior frontal	R	26	16	54	141	3.63
TD group						
Precuneus	L	-4	-68	54	102	3.98
Supramarginal	R	66	-44	24	163	3.80
Inferior parietal	R	62	-40	40	163	3.59
Inferior parietal	R	54	-42	22	163	3.35
Object > location						
ASD group						
Occipital pole	R	24	-94	-10	354	4.57
Lingual	R	24	-98	0	354	4.51
TD group						
Middle occipital	R	38	-92	6	109	6.53
Fusiform	L	-40	-48	-12	1,064	6.09
Inferior frontal (pars triangularis)	L	-54	22	10	335	4.92
Fusiform	R	38	-50	-22	648	4.81
Inferior frontal (pars triangularis)	L	-46	36	12	101	4.64
Medial frontal	L	-6	54	30	185	4.44
Lateral occipital	L	-36	-90	2	167	4.07
Lateral occipital	L	-42	-82	-2	167	3.24
Superior frontal	L	-12	40	50	93	3.94

*ASD* autism spectrum disorder, *TD* typically developing, *Hem* hemisphere.

**Figure 3:**
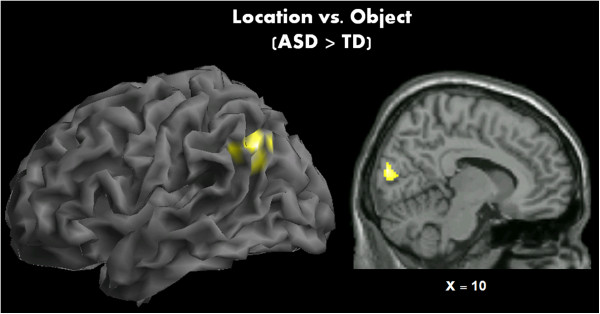
**Direct comparison between TD and ASD participants.** Children with ASD displayed significantly greater activation than TD controls during the location detection task.

Group differences in brain activation were determined by direct contrasts between TD and ASD children, which revealed significantly increased activity in left IPL (particularly in the angular gyrus, *k* = 146) and cuneus (*k* = 141) in ASD participants relative to TD participants during location detection (location > object) (see Figure 3). A similar increase in IPL activity was also seen in the contrast location > fixation (*k* = 247) in ASD participants, with some overlap with the middle temporal gyrus. There were no statistically significant group differences for object recognition; nor was there any significantly increased activity for TD participants relative to ASD participants.

### Functional connectivity

Based on previous findings of connectivity differences in ASD [10, 21, 22], we hypothesized intact functional connectivity in relatively posterior brain areas in our participants with ASD. Functional connectivity among individual ROIs revealed no statistically significant differences (after correcting for multiple comparisons) between ASD and TD participants in any of the ROI pairs. However, at an uncorrected statistical threshold, there was decreased connectivity in ASD participants in several pairs of ROIs during location detection, and between the ITG and SPL during object recognition, relative to TD participants (see Table 3 for the list of ROI pairings). In order to assess the validity of these results by adjusting for a large number of comparisons, the region-level functional connectivity analysis with 15 seed ROIs was followed up by a network connectivity analysis by grouping 12 of the ROIs into different networks (left parietal, right parietal, inferior temporal, occipital, and frontal) (see “Methods” section).

**Table 3:** **Functional connectivity between individual ROI pairs as well as functional connectivity network results for object recognition and location detection conditions**

	ASD		TD		
	Mean	SD	Mean	SD	*p*
ROI pair					
Object					
LITG : LSPL	0.37	0.37	0.68	0.25	0.02
LITG : RSPL	0.39	0.32	0.62	0.23	0.04
LITG : SMA	0.43	0.27	0.65	0.28	0.05
MPFC : RTHAL	0.49	0.25	0.83	0.40	0.03
ROC : RSPL	0.26	0.42	0.58	0.33	0.02
Location					
LITG : RITG	0.55	0.41	0.84	0.26	0.04
LMFG : MPFC	0.62	0.29	0.86	0.24	0.04
LMFG : SMA	0.74	0.32	1.00	0.28	0.04
MPFC : RTHAL	0.43	0.29	0.75	0.41	0.05
RIPL : SMA	0.48	0.43	0.83	0.25	0.03
RITG : ROC	0.33	0.36	0.64	0.35	0.02
RITG : RSPL	0.45	0.25	0.72	0.28	0.01
ROC : RSPL	0.35	0.38	0.62	0.32	0.04
FC Network					
Object					
LPAR : IT	0.43	0.23	0.60	0.23	0.04
Location					
RPAR : IT	0.47	0.31	0.68	0.21	0.04
IT : OCC	0.40	0.27	0.62	0.24	0.02

*LITG* left inferior-temporal gyrus, *LSPL* left superior parietal lobule, *RSPL* right superior parietal lobule, *SMA* supplementary motor area, *MPFC* medial prefrontal cortex, *RTHAL* right thalamus, *ROC* right occipital cortex, *LPAR* left parietal network, *OCC* occipital network.

There was decreased connectivity in ASD participants between the left parietal and inferior temporal networks (*p* = 0.04) during object recognition and between the inferior temporal and occipital network connections and the right parietal-to-inferior temporal connections in the location condition (*p* = 0.01 and *p* = 0.04, respectively). However, the network connectivity results for both conditions did not survive stepdown-Bonferroni corrections for multiple comparisons (initial *p* = 0.005) (see Table 3 for a list of significant ROI pairs and network pairs).

## Discussion

This study examined how dorsal and ventral visual streams respond to object recognition and location detection in ASD and TD children and adolescents. Both groups showed significantly increased activity in bilateral occipital and parietal areas during location detection. Parietal cortex is a critical area of the dorsal visual stream actively involved in visuospatial processing [29, 30] and visual attention [31, 32]. Locating the position of objects in the current study may involve several aspects of visual cognition. Object recognition, on the other hand, elicited ventral stream activation, especially the inferior temporal cortex in both groups. The inferior temporal cortex has been found to be actively involved in recognizing the shapes of objects [33, 34]. Single neuron recordings in macaque inferior temporal cortex [35] and human EEG [36] have shown image-specific responses as early as 100–150 ms after stimulus onset. Thus, while inferior temporal cortex is primarily involved in object recognition, inferior parietal is involved in location detection. Regarding group differences, the main finding of this study pertains to a significantly greater parietal activation in ASD participants, relative to TD, in the left hemisphere during the location detection task. In addition, the functional connectivity between frontal, temporal, and parietal areas displayed non-significant decreases in connectivity in individuals with ASD, suggesting that dorsal and ventral functional processes may be relatively intact. This is also evidenced from the behavioral results from our study which showed no significant group difference in reaction time or accuracy.

### Increased parietal cortex activation in ASD

Increased activation of the left inferior parietal cortex, a dorsal stream area, in individuals with ASD during location detection is consistent with similar findings from previous studies, especially with one of our own studies examining global and local processing in autism [21]. The peak of IPL activation reported in the current study is in the angular gyrus (AG), a region associated with multiple functions. It has a critical role in attention [37, 38], especially in the reorienting or shifting of attention [39]. The IPL, including the supramarginal gyrus and the AG, is part of a “bottom-up” attentional subsystem that mediates the automatic allocation of attention to task-relevant information [40], particularly in attending to retrieved memories [41]. It is possible that the participants in our study, especially ASD children, may associate the objects presented to their past experiences with it. The role of AG in spatial cognition is also important in the context of the current study. While IPL has been found to play a significant role in integrating information from dorsal and ventral streams [42], it has also been implicated in other functions, such as attention, praxis, self-other discrimination, visuospatial perception, and visualization of interaction with objects ([43–48]). The increased IPL activation is also interesting considering the somatosensory and attentional dysfunctions typically reported in individuals with ASD, and increased activity within this region could reflect a pattern of activation unique to individuals with ASD when processing or orienting to basic object stimuli.

Detecting the locations of objects or more “local” processing of objects has been argued to be intact to superior in ASD individuals, but such processing can be task-specific [49]. As such, the increased activity in the parietal cortex could reflect a difference in perceptual strategy used by ASD children for locating the position of objects. This could include a more local or “lower level” perceptual processing in accordance with theories of weak central coherence [50, 51] in autism, or it could reflect increased effort or difficulty in performing the tasks, which is suggested by prior studies finding difficulty in attending to (or failing to disengage from) objects presented in a visual field in individuals with ASD [52, 53]. Increased activation was also seen in cuneus in participants with ASD, relative to TD, while locating the position of objects. Cuneus has been found to act as a link between signals from striate and extrastriate cortices [54], suggesting the increased emphasis of ASD participants on visual coding in this task.

### Functional connectivity

While none of the decreases in connectivity in participants with ASD survived corrections for multiple comparisons, there were a noticeable number of decreased connections in autism between frontal, parietal, and inferior temporal regions during both tasks and decreased temporal-occipital connections during the location task. Studies of neuronal connectivity in monkeys have suggested that the IPL shares dense connections between several frontal (IFG, frontal eye fields, dorsolateral PFC), temporal (superior temporal, insula) somatosensory, and extrastriate visual areas. Thus, IPL may be a key region for visual and sensorimotor integration and response [55–57]. Thus, despite such widespread anatomical connections reported in literature (and increased activation in IPL seen in the current study), participants with ASD in our study showed decreased connectivity, perhaps suggesting the isolated and autonomous functioning of individual regions like IPL. A prominent model of visual processing suggests that the frontal cortex serves as a “top-down” mediator for processing stimuli between the ventral and dorsal streams in order to recognize objects [58]. The decreased connectivity between known targets of the IPL, coupled with the increased activation in extrastriate visual areas such as the cuneus in individuals with ASD during the location task, may necessitate alternate information processing strategies stemming from general frontal-posterior underconnectivity [26]. This disconnect of targets between visual processing streams may result in adaptive increases in activity to compensate for decreased communication between “bottom-up” (visual cortex, extrastriate and parietal regions) and “top-down” (frontal and temporal) regions of the brain in ASD. However, whether this disconnect could result in increased reliance on alternate perceptual strategies is uncertain.

## Conclusions

The findings of the present study support a relatively intact visuospatial processing in children with autism, albeit with minor decreases in frontal-posterior and temporal occipital-parietal connections in the brain. This may be due to the nature of neural (increased reliance on posterior cortical areas) and cognitive (less global and more detailed processing) information processing in this population. While intact or superior visual processing may be present in this population, increased parietal activation during location detection may underlie such advantage. It also suggests greater engagement of parietal/posterior areas in visuospatial tasks in general in autism, which may be the cause or consequence of decreased frontal-posterior connectivity. Future studies that focus on different modes of information processing (visuospatial, cognitive, social) in the same group of individuals can shed more light on the level and the extent of brain connectivity differences in autism.

## Electronic supplementary material

Additional file 1: **Sample representation of the regions of interest used in functional connectivity analysis.** A total of 15 ROIs were defined which included the following: the supplementary motor area (SMA), bilateral inferior parietal lobule (LIPL, RIPL), thalamus (LTHAL, RTHAL), inferior temporal gyrus (LITG, RITG), superior parietal lobule (LSPL, RSPL), occipital cortex (LOC, ROC), left middle frontal gyrus (LMFG), left precentral gyrus (LPRCN), medial prefrontal cortex (MPFC), and right hippocampus (RHIP). (DOCX 29 KB)

Additional file 2: **Activation peaks in ASD and TD groups for Location vs. Fixation, and Object vs. Fixation contrasts.** The tables give a detailed list of regions activated with cluster size for the given contrasts. (TIFF 326 KB)

## Abbreviations

ASD: autism spectrum disorder
FWHM: full width half maximum
IPL: inferior parietal lobe
ITG: inferior temporal gyrus
LIPL: left inferior parietal lobule
LITG: left inferior temporal gyrus
LMFG: left middle frontal gyrus
LOC: left occipital cortex
LPRCN: left precentral gyrus
LSPL: left superior parietal lobule
LSMA: left supplementary motor area
LTHAL: left thalamus
MPFC: medial prefrontal cortex
ROI: region of interest
RHIP: right hippocampus
RIPL: right inferior parietal lobule
RITG: right inferior temporal gyrus
ROC: right occipital cortex
RSPL: right superior parietal lobule
RTHAL: right thalamus
SPL: superior parietal lobe
SMA: supplementary motor area
TD: typically developing
WASI: Wechsler Abbreviated Scale of Intelligence.
