# Supplementary material for: Enhanced parietal cortex activation during location detection in children with autism
Source: J Neurodev Disord. 2014 Sep 19;6(1):37. doi: 10.1186/1866-1955-6-37 (PMC4190580; doi:10.1186/1866-1955-6-37)
Supplement: Supplementary file 1 — Additional file 1: Sample representation of the regions of interest used in functional connectivity analysis. A total of 15 ROIs were defined which included the following: the supplementary motor area (SMA), bilateral inferior parietal lobule (LIPL, RIPL), thalamus (LTHAL, RTHAL), inferior temporal gyrus (LITG, RITG), superior parietal lobule (LSPL, RSPL), occipital cortex (LOC, ROC), left middle frontal gyrus (LMFG), left precentral gyrus (LPRCN), medial prefrontal cortex (MPFC), and right hippocampus (RHIP). (DOCX 29 KB) [file 11689_2014_90_MOESM1_ESM.docx]

**Supplementary Table 1.** Activation peaks in ASD and TD groups for Location vs. Fixation contrast.

| **LOCATION > FIXATION** |  |  |  |  |  |  |
| --- | --- | --- | --- | --- | --- | --- |
| **ASD Group** |  |  |  |  |  |  |
| **Region** | **Hem** | **x** | **y** | **z** | **Cluster** | **t-value** |
| Cuneus/Occipital Pole | R | 12 | -100 | 12 | 7860 | 10.20 |
| Occipital Pole | L | -10 | -102 | 10 | 7860 | 9.50 |
| Occipital Pole | R | 26 | -96 | 8 | 7860 | 8.33 |
| Superior/Inferior Parietal Lobule | L | -36 | -58 | 54 | 3418 | 7.90 |
| Lateral Occipital Cortex | L | -30 | -60 | 32 | 3418 | 6.47 |
| Postcentral Gyrus | L | -42 | -24 | 48 | 3418 | 5.65 |
| Thalamus | L | -22 | -30 | -2 | 423 | 6.32 |
| Thalamus | L | -18 | -26 | 12 | 423 | 4.00 |
| Superior Parietal Lobule | R | 36 | -60 | 56 | 3235 | 5.95 |
| Superior Parietal Lobule | R | 28 | -50 | 40 | 3235 | 5.45 |
| Inferior Parietal Lobule | R | 34 | -52 | 48 | 3235 | 5.24 |
| Thalamus | R | 26 | -28 | 0 | 647 | 5.65 |
| Thalamus | R | 20 | -28 | 10 | 647 | 4.91 |
| Thalamic | R | 34 | -32 | 6 | 647 | 4.32 |
| Brainstem | R | -6 | -30 | -12 | 539 | 5.62 |
| Areas I-IV of Cerebellum | L | -8 | -50 | -16 | 539 | 4.47 |
| Vermis 3 of Cerebellum | L | 4 | -40 | -16 | 539 | 4.17 |
| Middle Frontal Gyrus | R | -30 | -4 | 40 | 149 | 4.62 |
| Middle Frontal Gyrus | L | -26 | 2 | 54 | 149 | 2.98 |
| Precentral Gyrus | L | -40 | 4 | 34 | 155 | 4.25 |
| Precentral Gyrus | L | -50 | 4 | 38 | 155 | 3.46 |
| Supplementary Motor Area | L | -6 | 10 | 52 | 370 | 3.94 |
| Supplementary Motor Area | R | 2 | 16 | 52 | 370 | 3.93 |
| Medial Frontal Gyrus | R | 14 | 16 | 48 | 370 | 3.65 |
| Insula | R | 40 | 18 | 0 | 86 | 3.78 |
|  |  |  |  |  |  |  |
| **TD Group** |  |  |  |  |  |  |
| **Region** | **Hem** | **x** | **y** | **z** | **Cluster** | **t-value** |
| Occipital Pole | R | 8 | -96 | 0 | 9348 | 12.68 |
| Occipital Pole | R | 22 | -98 | 16 | 9348 | 8.92 |
| Lingual Gyrus | L | -12 | -98 | 0 | 9348 | 8.09 |
| Thalamus | R | 26 | -28 | -2 | 125 | 6.11 |
| Hippocampus? | R | 32 | -34 | 2 | 125 | 3.02 |
| Thalamus | R | -24 | -30 | -2 | 112 | 5.74 |
| Supplementary Motor Area | R | 6 | 22 | 48 | 675 | 5.66 |
| Supplementary Motor Area | L | -2 | 12 | 52 | 675 | 4.76 |
| Superior Frontal Gyrus | L | -22 | 2 | 54 | 283 | 4.66 |
| Superior Frontal Gyrus | L | -22 | 6 | 68 | 283 | 4.40 |
| Middle Frontal Gyrus | L | 30 | 10 | 58 | 239 | 3.86 |
| Middle Frontal Gyrus | R | 26 | 6 | 52 | 239 | 3.81 |
| Pecentral | R | 36 | -12 | 62 | 239 | 3.41 |
| Precentral | L | -46 | 6 | 36 | 306 | 3.78 |
| Precentral | L | -56 | 14 | 36 | 306 | 3.67 |
| Crus I of the Cerebellum | R | 42 | -66 | -22 | 98 | 3.64 |
| Fusiform | R | 42 | -60 | -12 | 98 | 3.40 |
| Fusiform | R | 30 | -54 | -22 | 98 | 3.26 |
|  |  |  |  |  |  |  |

**Supplementary Table 2.** Activation peaks in ASD and TD groups for Object vs. Fixation contrast.

| **OBJECT > FIXATION** |  |  |  |  |  |  |
| --- | --- | --- | --- | --- | --- | --- |
| **ASD Group** |  |  |  |  |  |  |
| **Region** | **Hem** | **x** | **y** | **z** | **Cluster** | **t-value** |
| Occipital Pole | R | 28 | -94 | 8 | 9424 | 11.69 |
| Occipital Pole | L | -20 | -100 | 8 | 9424 | 11.32 |
| Occipital Pole | L | -12 | -102 | 10 | 9424 | 10.84 |
| Hippocampus | L | -22 | -32 | -2 | 562 | 10.65 |
| Brainstem | L | -8 | -32 | -8 | 562 | 3.61 |
| Hippocampus | R | 32 | -32 | -2 | 309 | 7.96 |
| Superior Parietal Lobule | L | -32 | -60 | 56 | 1121 | 6.80 |
| Superior Parietal Lobule | L | -28 | -66 | 50 | 1121 | 5.47 |
| Lateral Occipital Cortex | L | -28 | -64 | 34 | 1121 | 5.13 |
| Postcentral | L | -42 | -22 | 54 | 203 | 4.77 |
| Precentral | L | -42 | 2 | 32 | 236 | 4.61 |
| Precentral | L | -56 | 10 | 38 | 236 | 3.08 |
| Inferior Frontal (Pars Triangularis) | L | -32 | 30 | 6 | 170 | 4.31 |
| Insula | L | -30 | 26 | -8 | 170 | 3.70 |
| Suplementary Motor Area | L | -4 | 10 | 54 | 177 | 4.18 |
| Suplementary Motor Area | R | 4 | 10 | 56 | 177 | 3.77 |
| Precentral | R | 44 | -18 | 60 | 86 | 3.65 |
| Precentral | R | 38 | -10 | 56 | 86 | 3.04 |
|  |  |  |  |  |  |  |
| **TD Group** |  |  |  |  |  |  |
| **Region** | **Hem** | **x** | **y** | **z** | **Cluster** | **t-value** |
| Occipital Pole | R | 28 | -94 | 8 | 9424 | 11.69 |
| Occipital Pole | L | -20 | -100 | 8 | 9424 | 11.32 |
| Occipital Pole | R | -12 | -102 | 10 | 9424 | 10.84 |
| Hippocampus | L | -22 | -32 | -2 | 562 | 10.65 |
| Brainstem | L | -8 | -32 | -8 | 562 | 3.61 |
| Hippocampus | R | 32 | -32 | -2 | 309 | 7.96 |
| Superior Parietal Lobule | L | -32 | -60 | 56 | 1121 | 6.80 |
| Superior Parietal Lobule | L | -28 | -66 | 50 | 1121 | 5.47 |
| Lateral Occipital Cortex | L | -28 | -64 | 34 | 1121 | 5.13 |
| Postcentral Gyrus | L | -42 | -22 | 54 | 203 | 4.77 |
| Postcentral Gyrus | L | -42 | 2 | 32 | 236 | 4.61 |
| Postcentral Gyrus | L | -56 | 10 | 38 | 236 | 3.08 |
| Inferior Frontal (Pars Triangularis) | L | -32 | 30 | 6 | 170 | 4.31 |
| Insula | L | -30 | 26 | -8 | 170 | 3.70 |
| Supplementary Motor Area | L | -4 | 10 | 54 | 177 | 4.18 |
| Supplementary Motor Area | R | 4 | 10 | 56 | 177 | 3.77 |
| Precentral Gyrus | R | 44 | -18 | 60 | 86 | 3.65 |
| Precentral Gyrus | R | 38 | -10 | 56 | 86 | 3.04 |
|  |  |  |  |  |  |  |
