# Supplementary figures and images for: Enhanced parietal cortex activation during location detection in children with autism
Source: J Neurodev Disord. 2014 Sep 19;6(1):37. doi: 10.1186/1866-1955-6-37 (PMC4190580; doi:10.1186/1866-1955-6-37)

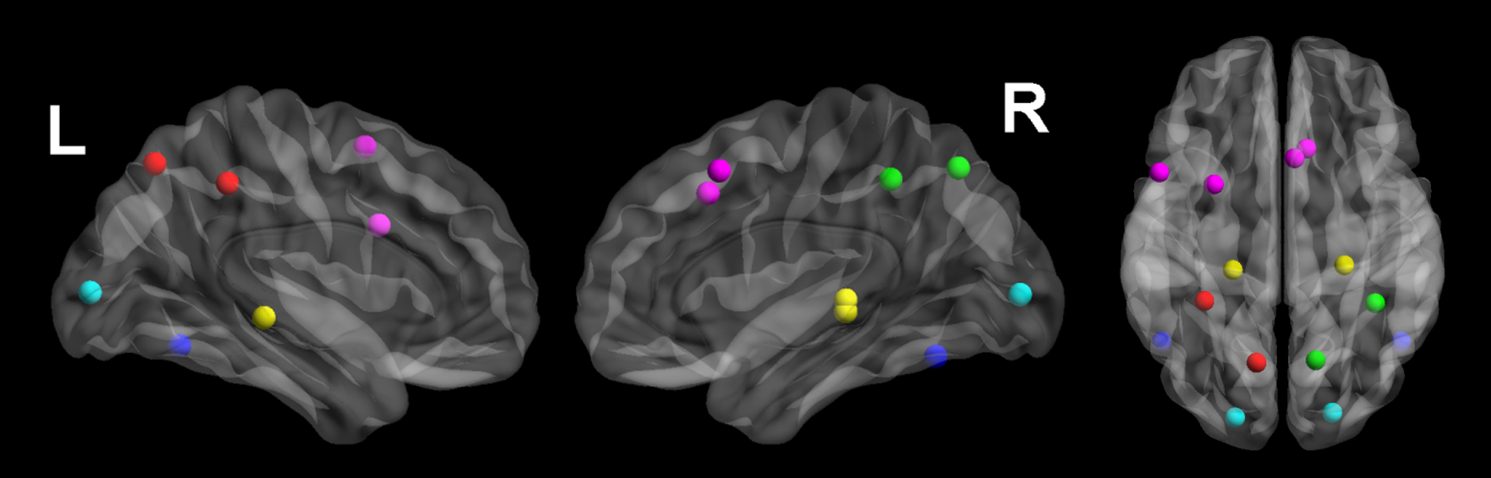

Supplement: Supplementary file 2 — Additional file 2: Activation peaks in ASD and TD groups for Location vs. Fixation, and Object vs. Fixation contrasts. The tables give a detailed list of regions activated with cluster size for the given contrasts. (TIFF 326 KB) [file 11689_2014_90_MOESM2_ESM.tiff]
